# Supplementary material for: Functional Segments on Intrinsically Disordered Regions in Disease-Related Proteins
Source: Biomolecules. 2019 Mar 5;9(3):88. doi: 10.3390/biom9030088 (PMC6468909; doi:10.3390/biom9030088)
Supplement: Supplementary file 1 [file biomolecules-09-00088-s001.zip › Anbo_FigureS1.pdf]

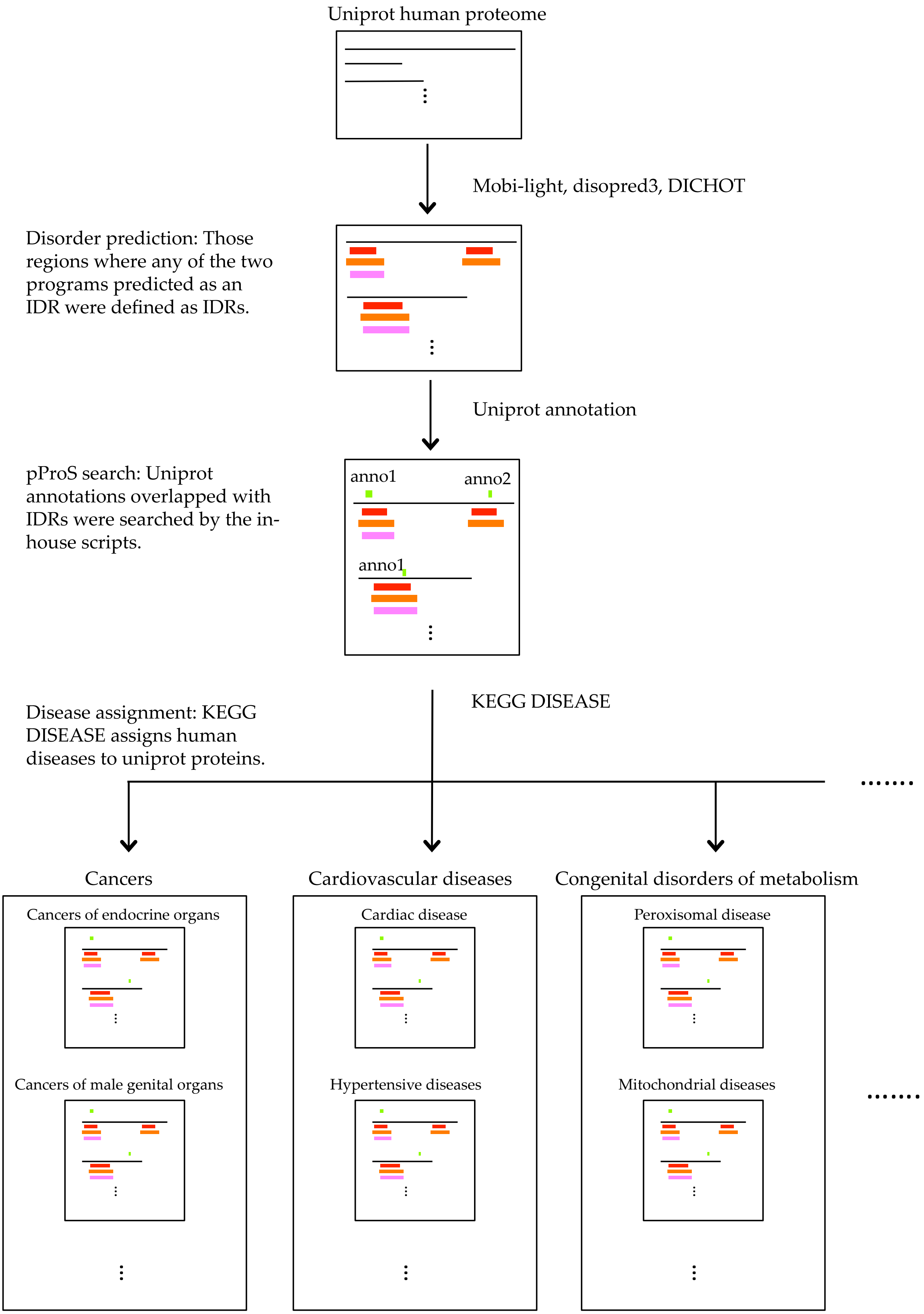
Uniprot human proteome
Mobi-light, disopred3, DICHOT
Disorder prediction: Those regions where any of the two programs predicted as an IDR were defined as IDRs.
Uniprot annotation
pProS search: Uniprot annotations overlapped with IDRs were searched by the in-house scripts.
anno1
anno2
anno1
KEGG DISEASE
Disease assignment: KEGG DISEASE assigns human diseases to uniprot proteins.
Cancers
Cancers of endocrine organs
Cancers of male genital organs
Cardiovascular diseases
Cardiac disease
Hypertensive diseases
Congenital disorders of metabolism
Peroxisomal disease
Mitochondrial diseases
