# Supplementary material for: Functional Segments on Intrinsically Disordered Regions in Disease-Related Proteins
Source: Biomolecules. 2019 Mar 5;9(3):88. doi: 10.3390/biom9030088 (PMC6468909; doi:10.3390/biom9030088)
Supplement: Supplementary file 1 [file biomolecules-09-00088-s001.zip › Anbo_FigureS2.pdf]

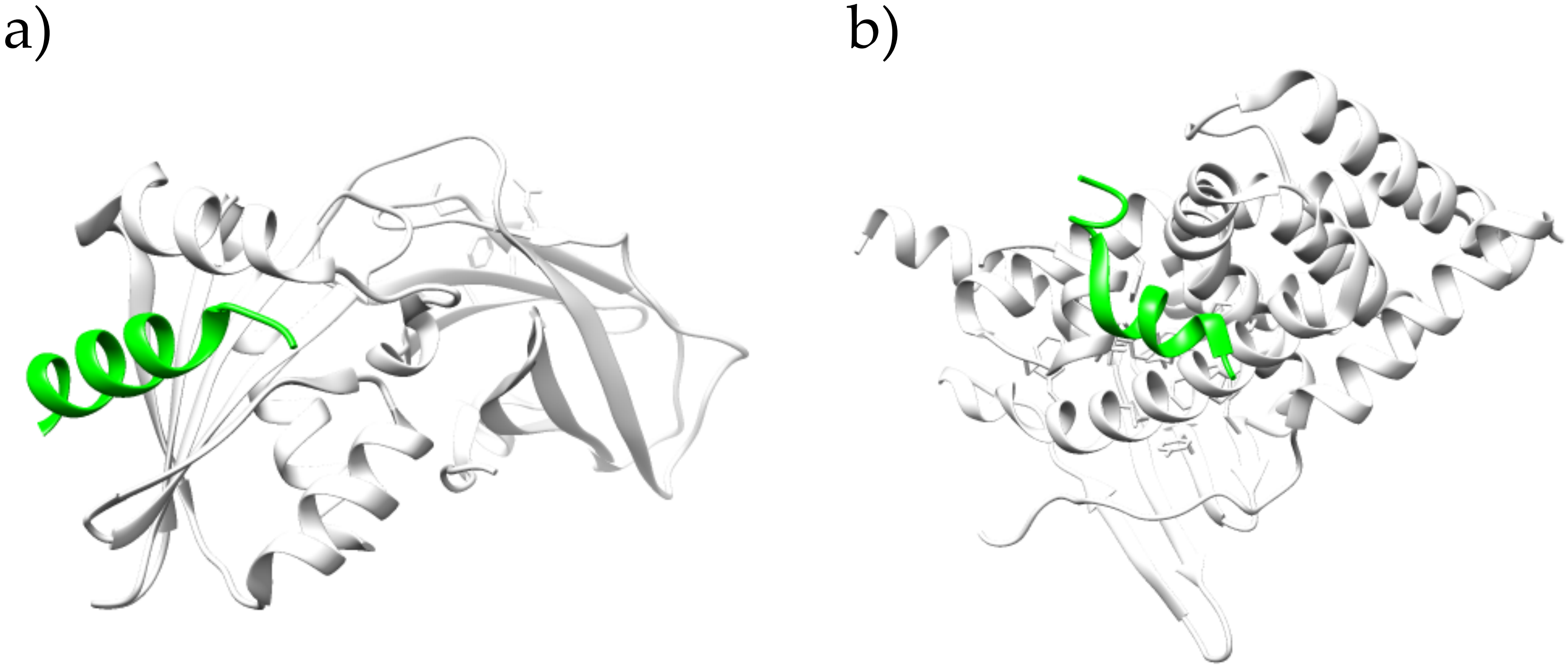

Figure S1. A structural example of a functional fragment found in an experimentally verified IDR. a) The structure of the AP-2 complex binding region (green) of phosphotyrosine- binding domain protein (ARH) binding upon AP-2 beta-subunit (white) (PDB: 2g30). B) A typical ProS structure of nuclear receptor co-activator 1 (green) (IDEAL: IID50084) binding upon nuclear receptor subfamily 1 group I (white).
