# Supplementary material for: Functional Segments on Intrinsically Disordered Regions in Disease-Related Proteins
Source: Biomolecules. 2019 Mar 5;9(3):88. doi: 10.3390/biom9030088 (PMC6468909; doi:10.3390/biom9030088)
Supplement: Supplementary file 1 [file biomolecules-09-00088-s001.zip › Anbo_FigureS3.pdf]

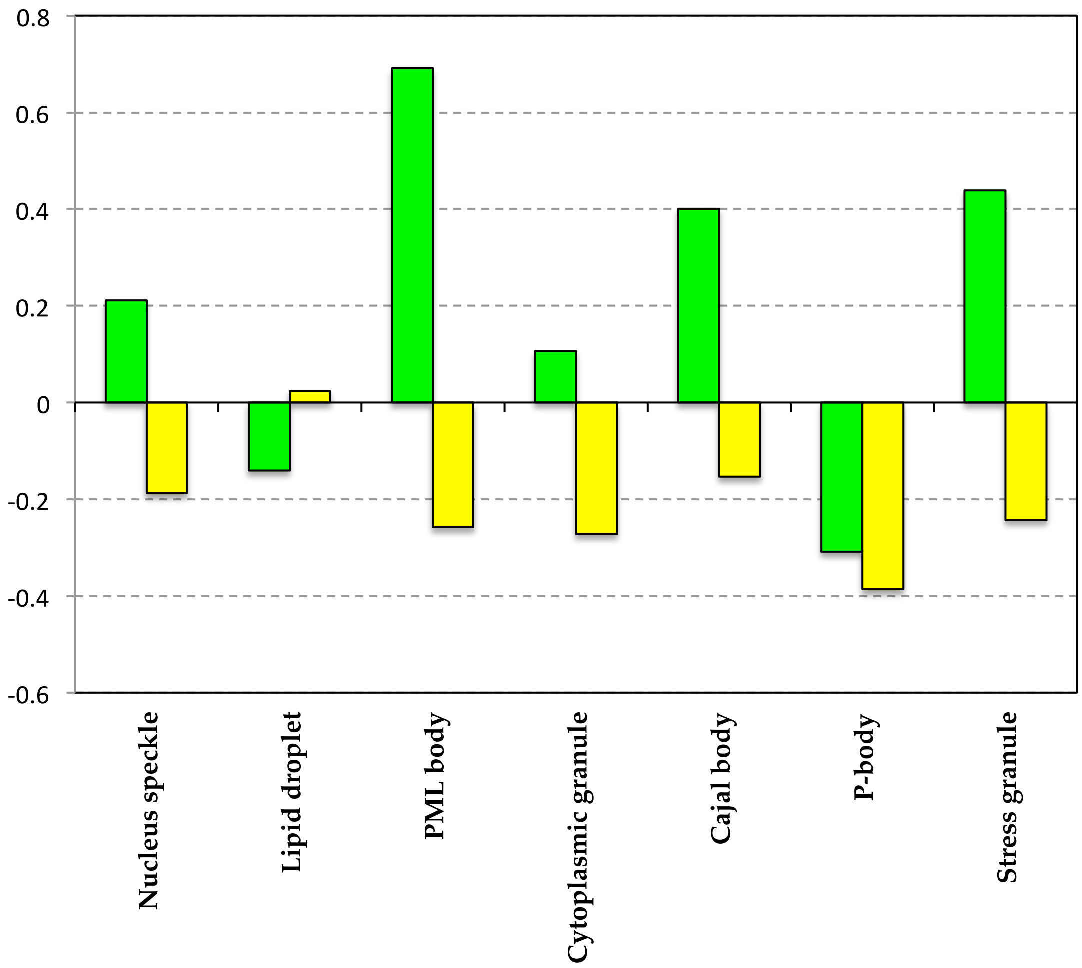

Figure S2. The non-membrane organelles found in the annotations of the disease-related proteins . The bars represent the degree of over-representation in each of the location categories, where green represents pProS-containing proteins, and yellow represents non-pProS proteins (see also Materials and Methods).
