# Supplementary material for: Functional Segments on Intrinsically Disordered Regions in Disease-Related Proteins
Source: Biomolecules. 2019 Mar 5;9(3):88. doi: 10.3390/biom9030088 (PMC6468909; doi:10.3390/biom9030088)
Supplement: Supplementary file 1 [file biomolecules-09-00088-s001.zip › Anbo_TableS1.pdf]

Table S1. pProSs frequently found in the disease-related proteins.

feature name	#counts	#proteins	description
Nuclear localization signal	92	83	sequence targeting to nucleus
Cell attachment site	49	18	cell adhesion related sequence
PDZ-binding	20	20	PDZ domain binding
LXXLL motif	16	11	essential for interaction with nuclear receptors
SH3-binding	15	12	SH3 domain binding
DNA-binding	14	7	DNA binding
Nuclear export signal	12	10	sequence exporting from nucleus
PPPSP motif	9	2	phosphorylation site
Abolishes interaction with PCNA.	8	2	sites inhibiting interaction with PCNA
Bipartite nuclear localization signal	8	8	sequence targeting to nucleus
Microbody targeting signal	8	8	sequence targeting microbody
Prevents secretion from ER	8	8	short segments preventing secretion
