# Supplementary material for: Functional Segments on Intrinsically Disordered Regions in Disease-Related Proteins
Source: Biomolecules. 2019 Mar 5;9(3):88. doi: 10.3390/biom9030088 (PMC6468909; doi:10.3390/biom9030088)
Supplement: Supplementary file 1 [file biomolecules-09-00088-s001.zip › Anbo_TableS2.pdf]

Table S2. The list of disease involving p53.

category	disease
Can	Adrenal carcinoma
Can	Adult T-cell leukemia
Can	Basal cell carcinoma
Can	Bladder cancer
Can	Breast cancer
Can	Burkitt lymphoma
Can	Cancer of the anal canal
Can	Cholangiocarcinoma
Can	Choriocarcinoma
Can	Chronic lymphocytic leukemia (CLL)
Can	Chronic myeloid leukemia (CML)
Can	Colorectal cancer
Can	Endometrial cancer
Can	Esophageal cancer
Can	Fallopian tube cancer
Can	Gallbladder cancer
Can	Gastric cancer
Can	Glioma
Can	Hairy-cell leukemia
Can	Hepatic angiosarcoma
Can	Hepatocellular carcinoma
Can	Kaposi sarcoma
Can	Laryngeal cancer
Can	Malignant pleural mesothelioma
Can	Mantle cell lymphoma
Can	Medulloblastoma
Can	Melanoma
Can	Merkel cell carcinoma
Can	Multiple myeloma
Can	Mycosis fungoides
Can	Non-small cell lung cancer
Can	Oral cancer
Can	Oropharyngeal cancer
Can	Osteosarcoma
Can	Ovarian cancer
Can	Pancreatic cancer
Can	Penile cancer
Can	Small cell lung cancer
Can	Squamous cell carcinoma
Can	Thyroid cancer
Can	Vulvar cancer
Car	Myelodysplastic syndrome
Mus	Giant cell tumor of bone
Ner	Choroid plexus papilloma
Oth	Li-Fraumeni syndrome
