# Supplementary material for: Functional Segments on Intrinsically Disordered Regions in Disease-Related Proteins
Source: Biomolecules. 2019 Mar 5;9(3):88. doi: 10.3390/biom9030088 (PMC6468909; doi:10.3390/biom9030088)
Supplement: Supplementary file 1 [file biomolecules-09-00088-s001.zip › Anbo_TableS4.pdf]

Table S4. Annotations found on IDRs in the model organisms

Organism	mutagenesis site	region of interest	short sequence motif
Mouse	1703	253	1160
Rat	620	161	575
A. thaliana	932	44	595
yeast	614	161	575

The predictions of IDRs were done only by DICHOT, and the annotations shorter than 10 residues were considered.
