# Supplementary material for: Functional Segments on Intrinsically Disordered Regions in Disease-Related Proteins
Source: Biomolecules. 2019 Mar 5;9(3):88. doi: 10.3390/biom9030088 (PMC6468909; doi:10.3390/biom9030088)
Supplement: Supplementary file 1 [file biomolecules-09-00088-s001.zip › Anbo_TableS5.pdf]

Table S5: The redundancy of pProS by the disease categories.

	pProS counts	#uniq pProSs	pProS redundancy	#proteins
Cancers	859	147	5.8	204
Cardiovascular diseases	144	93	1.5	335
Congenital disorders of metabolism	78	53	1.5	687
Congenital malformations	335	242	1.4	832
Digestive system diseases	32	32	1.0	79
Endocrine and metabolic diseases	90	63	1.4	213
Immune system diseases	73	56	1.3	256
Musculoskeletal diseases	129	69	1.9	149
Nervous system diseases	241	199	1.2	795
Other congenital disorders	44	39	1.1	91
Reproductive system diseases	24	21	1.1	63
Respiratory diseases	1	1	1.0	55
Skin diseases	26	22	1.2	104
Urinary system diseases	33	33	1.0	66
Other diseases	84	68	1.2	194
