# Supplementary material for: Functional Segments on Intrinsically Disordered Regions in Disease-Related Proteins
Source: Biomolecules. 2019 Mar 5;9(3):88. doi: 10.3390/biom9030088 (PMC6468909; doi:10.3390/biom9030088)
Supplement: Supplementary file 1 [file biomolecules-09-00088-s001.zip › Anbo_TableS6.pdf]

Table S6: Proteins involved in multiple diseases.

protein name	Uniprot accession	#disease annotated	content of disease annotaion
Cellular tumor antigen p53	P04637	46	Can(42),Car(1),Mus(1),Ner(1),Oth(1)
Cyclin-dependent kinase inhibitor 2A	P42771	27	Can(26), End(1)
GTPase KRas	P01116	22	Can(18), Mal(4)
Myc proto-oncogene protein	P01106	18	Can(18)
HLA class II histocompatibility antigen, DRB1-3 chain	P01912	17	Car(3), End(1), Imm(9), Ner(2), Ski(2)
Phosphatidylinositol 3,4,5-trisphosphate 3-phosphatase and dual-specificity protein phosphatase PTEN	P60484	14	Can(11), Mal(3)
Collagen alpha-1(II) chain	P02458	13	Mal(9), Mus(3), Ner(1)
HLA class II histocompatibility antigen, DQ beta 1 chain	P01920	13	Car(2), Dig(1), End(1), Imm(6), Ner(1), Ski(2)
GTPase NRas	P01111	12	Can(8), Car(1), Mal(2), Imm(1)
Fibroblast growth factor receptor 3	P22607	12	Can(2), Mal(10)
